# Multi-Omics Identification of UBE2C as a Prognostic Biomarker and Therapeutic Target Linked to Topotecan Sensitivity in Cervical Cancer

**DOI:** 10.32604/or.2026.079551

**Published:** 2026-08-13

**Authors:** Emmanuel Naveen Raj, Chia-Jung Li, Shih-Hsuan Cheng, Su-Boon Yong, Zhi-Hong Wen, An-Jen Chiang

**Affiliations:** 1 Department of Obstetrics and Gynecology, Kaohsiung Veterans General Hospital, Kaohsiung, Taiwan; 2 Institute of BioPharmaceutical Sciences, National Sun Yat-sen University, Kaohsiung, Taiwan; 3 National Museum of Marine Biology & Aquarium, Pingtung, Taiwan; 4 Center of General Education, Cheng Shiu University, Kaohsiung, Taiwan; 5 Center of General Education, Shu-Zen Junior College of Medicine and Management, Kaohsiung, Taiwan; 6 Department of Allergy and Immunology, China Medical University Children’s Hospital, Taichung, Taiwan; 7 Research Center for Allergy, Immunology, and Microbiome (A.I.M.), China Medical University Hospital, Taichung, Taiwan; 8 Department of Marine Biotechnology and Resources, National Sun Yat-sen University, Kaohsiung, Taiwan; 9 Department of Medical Education and Research, Kaohsiung Veterans General Hospital, Kaohsiung, Taiwan

**Keywords:** UBE2C, tumor-associated macrophages, immune infiltration, cervical cancer, scRNA-seq, spatial transcriptomics, pharmacogenomics

## Abstract

**Objectives**: Cervical squamous cell carcinoma and endocervical adenocarcinoma (CESC) necessitate the discovery of novel biomarkers for prognostic and therapeutic advancement. This study aims to evaluate the clinical significance of ubiquitin-conjugating enzyme E2C (UBE2C) and its association with the tumor microenvironment (TME) in CESC. **Methods**: We meticulously sourced CESC data from renowned repositories such as The Cancer Genome Atlas (TCGA), Genotype-Tissue Expression (GTEx), and Gene Expression Omnibus (GEO), leveraging cutting-edge techniques including single-cell RNA sequencing (scRNA-seq), spatial transcriptomics, and pharmacogenomics. Through multifaceted data analysis, we endeavored to unravel the intricate role and potential value of UBE2C in CESC tumorigenesis and progression. **Results**: Analysis of public datasets confirms UBE2C elevation in CESC tumors, correlating with advanced stages, metastasis, and poor disease-free survival (DFS). Dependency screens and functional enrichment highlight UBE2C’s critical role in cell viability and DNA replication. Notably, multi-omics and spatial transcriptomics reveal a strong link between UBE2C expression and macrophage infiltration (CD63^+^) in tumor regions. Finally, pharmacogenomic profiling and molecular docking identified Topotecan as a potent therapeutic agent with high UBE2C binding affinity. **Conclusion**: In conclusion, UBE2C expression is associated with cervical cancer progression and correlates with an immunosuppressive macrophage-enriched microenvironment, making it a promising candidate for further investigation in therapeutic intervention.

## 1 Introduction

Cervical cancer remains a major malignancy within the female reproductive system and continues to contribute significantly to cancer-related deaths globally [1,2]. While widespread human papillomavirus vaccination and cytological screening have reduced the incidence in many developed regions, the prognosis for patients presenting with advanced stage or metastatic disease remains poor [3]. Current clinical management relies primarily on surgery, radiotherapy, and cisplatin-based chemotherapy, yet the molecular drivers underlying treatment resistance and aggressive progression are not fully understood [3,4]. Identifying novel biomarkers that can predict clinical outcomes and serve as potential therapeutic targets is therefore critical for improving patient care in this population.

The ubiquitin-proteasome system is a fundamental mechanism for maintaining protein homeostasis and regulating essential cellular functions, including the cell cycle [5]. Within this system, UBE2C acts as a pivotal regulator of mitotic exit [4,6]. UBE2C coordinates with the anaphase-promoting complex to facilitate the polyubiquitination and subsequent degradation of key mitotic regulators such as Cyclin B and securin, thereby ensuring the timely transition from metaphase to anaphase [7]. The dysregulation or overexpression of UBE2C has been implicated in the oncogenic transformation of various human tissues, leading to uncontrolled proliferation and genomic instability [5,8,9,10]. Previously, overexpression and knockdown of UBE2C enhanced and reduced cervical cancer cell proliferation and positively modulated by the mTOR/PI3K/AKT pathway [11]. Administration of UBE2C partially blunted the salutary effects of miR-525-5p on epithelial-mesenchymal transition (EMT), metastasis, and anoikis resistance in cervical cancer via blocking the UBE2C/ZEB1/2 signaling axis [12]. Moreover, vorinostat is a histone deacetylase inhibitor targets UBE2C, which reverses EMT and regulates cervical cancer cell proliferation through the ubiquitination pathway [13]. Recently, deficiency of UBE2C protein levels during cervical carcinogenesis modulates the glycolysis-associated protein F264, which partially activates tumor dormancy mechanisms by contributing to the intrinsic radioresistance in cervical cancer patients [14].

Analysis of large-scale clinical datasets demonstrates that UBE2C is significantly overexpressed in CESC tumor tissues compared to non-tumor controls. This elevated expression correlates with advanced cancer stages and the presence of metastasis, marking it as a critical marker of disease severity. Genomic profiling of these patients reveals a distinct mutational landscape where the UBE2C high group is characterized by high mutation frequencies in genes such as TTN, PIK3CA, and KMT2C. These findings suggest that UBE2C is not only a marker of progression but is also associated with a more complex genomic profile in cervical cancer.

Survival analysis indicates that patients with high UBE2C expression levels exhibit significantly shorter overall survival (OS) and disease-free survival (DFS) compared to those with low expression. Cox regression analysis across multiple datasets confirms that UBE2C serves as a robust prognostic variable. Furthermore, dependency screens performed across eighteen cervical cancer cell lines, including HeLa and CaSki, demonstrate that these malignant cells are highly dependent on UBE2C for survival. These results reinforce the status of UBE2C as a functional driver of malignancy and a potential target for therapeutic intervention [6,15].

The tumor microenvironment (TME) is a complex and dynamic ecosystem where malignant cells interact with a variety of stromal and immune populations [16]. Among these, tumor-associated macrophages (TAMs) are known to be highly plastic and can be polarized into phenotypes that either inhibit or promote tumor progression [17]. In many solid tumors, macrophages are recruited to the tumor site and reprogrammed to support an immunosuppressive niche that facilitates immune evasion and angiogenesis [7]. Recent studies have begun to explore how tumor cell intrinsic genetic alterations can influence the recruitment and functional polarization of macrophages within the local microenvironment [11,18].

Using multiple deconvolution algorithms such as CIBERSORT and xCell, a robust positive correlation has been identified between UBE2C expression and the infiltration levels of macrophages in cervical cancer, particularly M2 and TAMs. scRNA-seq further reveals that UBE2C is expressed both in malignant clusters and specific macrophage populations, particularly within the C19 cluster. These findings suggest that UBE2C high tumor cells may be responsible for orchestrating the recruitment and functional reprogramming of macrophages, thereby contributing to the development of a pro-tumor immunological landscape.

The advent of high-resolution spatial transcriptomics has allowed for the detailed mapping of tissue architecture and cellular neighborhoods [18]. Spatial transcriptomic profiling using 10× Visium technology identifies distinct functional domains within the cervical cancer tissue. The tissue can be partitioned into advanced and tumor regions based on the enrichment of biomarkers such as S100A8 and KRT13. Specifically, spatial co-localization analysis identifies hotspots where high UBE2C expression overlaps with TAMs markers such as CD63 and cell cycle markers such as CDC20 in the advanced tumor regions. This spatial concordance provides strong evidence for a shared niche or direct communication between UBE2C-high tumor cells and TAMs and cell cycle progression.

Mechanistically, UBE2C is intrinsically linked to biological processes involving DNA-dependent DNA replication and mitotic organization. Silencing of UBE2C leads to a significant downregulation of mitotic regulators such as Cyclin B1 also known as CCNB1, and CDC25C, emphasizing its necessity for maintaining the proliferative capacity of cervical cancer cells. Given its dual role in driving proliferation and immune remodeling, UBE2C represents an attractive therapeutic target. Pharmacogenomic analysis and molecular docking simulations identify topotecan as a potential small-molecule inhibitor with high binding affinity to the UBE2C protein structures 1I7K and 4YII.

The primary objective of this study is to provide a comprehensive characterization of the UBE2C landscape in CESC by integrating population-level clinical data with high resolution spatial and single cell transcriptomics. We aim to elucidate the molecular mechanisms by which UBE2C drives tumor progression and influences the recruitment and activation of TAMs. Furthermore, we seek to validate the therapeutic potential of targeting the UBE2C axis through pharmacogenetic profiling. By establishing the spatial and molecular links between UBE2C and the immune microenvironment, this research provides a deeper understanding of cervical cancer pathogenesis and identifies new avenues for personalized treatment strategies.

## 2 Materials and Methods

### 2.1 Study Design and Data Integration

The present work was carried out as a retrospective analysis that combined multi-omics data mining with experimental validation using clinical samples. Publicly available resources, including bulk RNA sequencing, scRNA-seq, and spatial transcriptomic datasets, were systematically examined to characterize patterns of UBE2C expression and its relationship to the tumor immune microenvironment (TIME). To complement these computational analyses, immunofluorescence and western blot analysis were performed in cervical cancer cells, providing additional evidence at the protein level. Together, these approaches allowed us to explore not only the clinical and molecular relevance of UBE2C but also its potential pharmacogenomic associations in CESC.

### 2.2 Multi-Omics Analysis

Multi-omics analysis of UBE2C in CESC encompasses the utilization of diverse bioinformatics tools and platforms. Data from diverse platforms including The University of Alabama at Birmingham CANcer (UALCAN) portal (http://ualcan.path.uab.edu) [19], Gene expression omnibus (GEO) database (https://www.ncbi.nlm.nih.gov/geo/) [20], The Cancer Genome Atlas (TCGA) database (https://portal.gdc.cancer.gov/) [21], Genotype-Tissue Expression (GTEx) portal (https://www.gtexportal.org/home/) [22], Comprehensive Analysis on Multi-Omics of Immunotherapy in Pan-cancer (CAMOIP) (http://www.zjyy-oncology.com:20002/) [23], Biomarker Exploration for Solid Tumors (BEST) (https://rookieutopia.hiplot.com.cn/app_direct/BEST/) [24], Tumor Immune Estimation Resource 3 (TIMER3) (https://compbio.cn/timer3/) [25], The Tumor Immune Single Cell Hub 2 (TISCH2) (http://tisch.comp-genomics.org/) [26], GeneMANIA (http://www.genemania.org) [27], LinkedOmics (https://www.linkedomics.org/admin.php) [28], Dependency Map (DepMap) (https://depmap.org) [29], Metascape (https://metascape.org/gp/index.html#/main/step1) [30], Comprehensive Pan-cancer analysis of Drug sensitivity (CPADS) (https://smuonco.shinyapps.io/CPADS/) [31], and Q-omics (https://qomics.ai/) [32], were harnessed to assess the gene mutations landscape, gene expression, clinical features (types, tumor stages and metastasis), survival analysis, CRISPR cancer cell line screening, immune infiltration, protein-protein interactions, positive and negative correlation of genes, gene-set enrichment analysis, functional enrichment analysis, single-cell transcriptome analysis and drug analysis.

### 2.3 Single-Cell Data Processing and Immune Profiling

We downloaded the scRNA-seq dataset of cervical cancer (GSE168652) along with the annotation information from the TISCH2 database (http://tisch.comp-genomics.org/) and reannotated and visualized it using the “Seurat” R package (version 4.3.0). Specifically, cells were filtered based on the following criteria: (1) genes detected per cell were required to be between 200 and 6000 to exclude cell fragments and doublets; (2) the total UMI count per cell was limited to a minimum of 500; and (3) the percentage of mitochondrial gene expression was restricted to less than 20% to ensure cell viability. After QC filtering, the data were normalized and scaled. Variable features were identified, and Principal Component Analysis (PCA) was performed for initial linear dimensionality reduction. Based on the elbow plot, the top 30 principal components (PCs) were selected for further downstream analysis. For non-linear visualization, Uniform Manifold Approximation and Projection (UMAP) was implemented using the first 30 PCs as input. Cell clustering was performed using the FindNeighbors function (based on 30 neighbors) and the FindClusters function with a resolution set to 0.8 to determine optimal cluster granularity. This integrated pipeline allowed for the precise identification of malignant cells and specific immune deconvolution within the TME.

### 2.4 Immune Deconvolution and Algorithmic Integration

To robustly assess the correlation between UBE2C expression and immune cell infiltration, we utilized the immunedeconv R package (version 2.1.0), which integrates multiple computational algorithms. Specifically, the TIMER3 (https://compbio.cn/timer3/) “Gene” module was employed to evaluate the relationship between UBE2C and various immune cell types, using the Pearson correlation method to determine statistical associations.

To ensure the consistency of our findings across different computational models, we further applied CIBERSORT (with 100 permutations), Quantiseq, and xCell algorithms. For each method, default key parameters recommended by the developers were maintained. The results from these diverse algorithms were integrated through a consensus-based approach; a correlation was considered biologically significant only if it was consistently identified as significant (*p < 0.05) across at least three distinct deconvolution methods. This multi-algorithmic comparison was used to minimize tool-specific biases and validate the strong positive correlation between UBE2C and macrophage infiltration.

### 2.5 Spatial Transcriptomics Analysis

Spatial transcriptomic datasets from previously published studies were analyzed by summarizing unique molecular identifiers (UMIs) within predefined spatial bins. Clusters were then annotated with reference to hematoxylin and eosin (H&E) staining to align transcriptomic features with tissue morphology. We specifically examined the spatial distribution of UBE2C together with oncogenic/pro-tumor, tumor suppressive, and macrophage related genes such as S100A8, VEGFA, KRT13, TPM2, CDC20, and CD63.

### 2.6 Immune Landscape Characterization Using ssGSEA

To further characterize the immune landscape, single-sample gene set enrichment analysis (ssGSEA) was implemented using the GSVA R package (version 1.46.0) to estimate the immune cell composition within each spatial cluster. The enrichment scores were calculated based on established immune cell-specific gene sets derived from the Molecular Signatures Database (MSigDB) and literature-validated markers. For the ssGSEA parameters, the kernel estimation was set to ‘Gaussian’ (default for log-normalized data), and the enrichment scores were normalized to range between 0 and 1. This standardized approach allowed for a robust quantitative comparison of immune cell infiltration across different spatial regions.

### 2.7 Human Tissue Samples

Human tissue samples and associated clinical data were obtained from the KSVGH Biobank under approval from the Biobank Ethics Governance Council (Protocol No. KSVGH22-003) and the Ethics Governance Committee (Protocol No. KSVGH21-008). Informed consent was obtained from all individual participants included in the study. All procedures performed in this study were in accordance with the ethical standards of the institutional research committee and with the 1964 Declaration of Helsinki and its later amendments or comparable ethical standards.

### 2.8 Ethic Statement

This study utilized specimens and associated clinical data, including patient history and cancer stage, retrieved from the Kaohsiung Veterans General Hospital (KSVGH) Biobank. All clinical investigations were conducted in accordance with the approvals from the Biobank Ethics Governance Council of KSVGH (Protocol No. KSVGH22-003). The release and utilization of these human cervical cancer materials were further approved by the respective Ethics Governance Committees under protocol KSVGH21-008. All procedures performed in this study were in accordance with the ethical standards of the institutional research committee and with the 1964 Declaration of Helsinki and its later amendments or comparable ethical standards. Informed consent was obtained from all individual participants included in the study. All specimens and data were provided in a strictly anonymized form to ensure patient confidentiality and privacy.

### 2.9 RNA Extraction and Real-Time Polymerase Chain Reaction (PCR)

Total RNA was extracted from collected cervical specimens (approximately 20 mg of tissue per sample) using the EasyPrep Total RNA Kit (Biotools, Taipei, Taiwan; Cat. No. DP201) according to the manufacturer’s instructions. RNA concentration and purity were assessed using a NanoDrop 2000 Spectrophotometer (Thermo Fisher Scientific, Wilmington, DE, USA), ensuring an A260/A280 ratio between 1.8 and 2.0 prior to downstream applications. Subsequently, cDNA was synthesized from 1 μg of the extracted total RNA in a 20 μL reaction volume using the ToolScript MMLV RT Kit (Biotools, Taipei, Taiwan; Cat. No. TT-RT02) and TOOLS M-MLV RTase (Biotools, Taipei, Taiwan; Cat. No. TT-RT03). The thermal cycling conditions for reverse transcription were: 25°C for 10 min, 42°C for 50 min, and 70°C for 15 min. Quantitative real-time PCR (qPCR) analysis was performed using the StepOnePlus Real-Time PCR System (Applied Biosystems, Foster City, CA, USA; Model No. 2720). Each 20 μL qPCR reaction contained 10 μL of 2X SYBR Green Master Mix (Biotools, Taipei, Taiwan; Cat. No. TT-K11), 0.5 μM of each forward and reverse primer, and 2 μL of diluted cDNA. The cycling conditions involved an initial denaturation at 95°C for 10 min, followed by 40 cycles of 95°C for 15 s and 60°C for 1 min. The specific primer sequences used were: UBE2C Forward: 5′-TGGTGGGCCTAGATGAAGAC-3′, Reverse: 5′-CTGCTGTAGCCAGATCCACA-3′; and U6 (as an internal control) Forward: 5′-CTCGCTTCGGCAGCACATATACT-3′, Reverse: 5′-ACGCTTCACGAATTTGCGTGTC-3′. Relative gene expression levels were determined using the 2^−∆∆Ct^ method, with U6 used as the normalization strategy. All nucleic acid reagents and kits utilized in these procedures were purchased from Biotools (Taipei, Taiwan).

### 2.10 Cells and Reagents

In this study, multiple human cervical cancer cell lines, including HeLa cells (BCRC#60005, Hsinchu, Taiwan), and CC7T/VGH cells (BCRC#60195, Hsinchu, Taiwan), were purchased from the Bioresource Collection and Research Center (BCRC, Hsinchu, Taiwan). The CaSki cell line (ATCC#CRL-1550, Manassas, VA, USA) was obtained from the American Type Culture Collection (ATCC, Manassas, VA, USA). All cell lines used in this study were authenticated by the respective sources using Short Tandem Repeat (STR) profiling and were confirmed to be negative for mycoplasma contamination via regular testing based on BCRC, and ATCC protocols. Cells were cultured in DMEM (Thermo Fisher Scientific, Waltham, MA, USA; Cat. No. 11965092) supplemented with 10% FBS (Thermo Fisher Scientific, Waltham, MA, USA; Cat. No. 10437028) in a humidified atmosphere of 95% air and 5% CO_2_ at 37°C as described previously [11]. Topotecan drug was purchased from Cayman Chemical (Sigma-Aldrich, Inc., St. Louis, MO, USA; Cat. No. MHS32).

### 2.11 Human Tissue Microarray (TMA) Immunohistochemical Analysis

Tumor tissues from formalin-fixed, paraffin-embedded tissue blocks of carcinomas with a core size of 1.5 mm were assessed. Sampling sites including 2 tumor sites and 1 non-tumor site were marked on each donor block by a pathology physician, and the tissue cylinders were precisely arrayed into recipient blocks, each with a core size of 1.5 mm. The blocks of embedded tissue for TMA were performed using a manual tissue microarrayer (Beecher Instruments, Silver Spring, MD, USA); and the recipient was incubated overnight at 37°C before sectioning. Immunohistochemical analysis in the TMA sections was carried out as described in a previous study [11,33]. The TMA sections (4 μm) were deparaffinized in xylene, slides were dehydrated in an alcohol graded series and placed in running water. The Novolink Polymer Detection System (Leica Microsystems Inc., Newcastle Upon Tyne, UK; Cat. No. RE2780-CE) was used for immunohistochemistry. The antigen was retrieved with heating in 10 mM citrate buffer (pH 6.0) (Sigma, St. Louis, MI, USA; Cat. No. C9999) and slides were incubated with Peroxidase Block to neutralize endogenous peroxidase activity and then slides were incubated with 10 μg/mL proteinase K (Sigma-Aldrich, St. Louis, MO, USA; Cat. No. 39450016) at 37°C for 30 min. Then the slides Protein Block before reaction with anti-UBE2C antibody (Abnova, Taipei, Taiwan; Cat. No. H00011065-M01; 1:100 dilution). Then, slides were reacted with Novolink Polymer followed by 3,3′-diaminobenzidine-tetrahydrochloride (DAB) chromogen solution (Sigma, St. Louis, MI, USA; Cat. No. D7304) to develop peroxidase activity for visualizing the antibody-hydrochloride complex, and slides were counterstained with hematoxylin (Sigma-Aldrich, Inc., St. Louis, MO, USA; Cat. No. MHS32).

### 2.12 Evaluation of Human TMA Sections

The IHC-stained TMA sections were evaluated for UBE2C expression by assessing both the percentage of positively stained tumor cells (0–100%) and the staining intensity. Staining intensity was categorized as negative (–), weak (+), moderate (++), or strong (+++). A histological score (H-score), ranging from 0 to 300, was then calculated to provide a quantitative assessment of UBE2C protein levels using the following formula: H-score = [1 × (% cells 1+) + 2 × (% cells 2+) + 3 × (% cells 3+)]. To ensure objectivity and minimize bias, the H-score evaluation was performed independently by two experienced pathologists in a blinded manner, with no prior knowledge of the patients’ clinical characteristics. Any discrepancies in scoring between the two observers were resolved through discussion and consensus using a multi-headed microscope to minimize inter-observer variability. Samples that were damaged or contained insufficient tumor tissue (less than 10% of the core area) were excluded from the final analysis to maintain data integrity. For subsequent statistical analysis and prognostic correlation, the tumors were classified into three groups based on their H-scores: 0–100, 101–200, and 201–300. The Kruskal-Wallis test followed by Dunn’s post-hoc test was used to compare UBE2C levels across different clinical cancer stages (I–IV) in our cohort of 167 patient samples. All IHC evaluations and subsequent *in vitro* experiments were performed in at least three independent biological replicates, with appropriate negative and positive controls included in each run to ensure experimental reliability.

### 2.13 Cell Proliferation Assay

Cell proliferation was evaluated using the colorimetric WST-1 assay (Roche Applied Science, Indianapolis, IN, USA; Cat. No. 11644807001) according to the manufacturer’s instructions. The WST-1 assays were used to monitor the cell proliferation of HeLa cells. Briefly, untreated controls and UBE2C shRNA knockdown cells were trypsinized and resuspended in culture medium, then plated 5 × 10^3^ cells per well in 96-well plates in triplicate and incubated for 24, 48, and 72 h. The cells were then incubated with 10 μL of WST-1 reagent for 2 h. The absorbance at 450 nm was monitored, and the reference wavelength was set at 620 nm. The absorbance for WST-1 proliferation assays was measured using a Multiskan FC Microplate Photometer (Thermo Fisher Scientific, Waltham, MA, USA; Model No. 51119100). Each condition was assessed in triplicate, and the entire experiment was independently repeated three times to ensure technical and biological reproducibility.

### 2.14 shRNA-Mediated Knockdown of UBE2C in HeLa Cells

To silence UBE2C expression, HeLa cells were seeded at a density of 2 × 10^5^ cells per well in 6-well culture plates. Transfection was performed using UBE2C-specific shRNA plasmids (Target sequence: 5′-GGUGCUGCUCCGGCUUAUUTT-3′; Sigma-Aldrich, St. Louis, MO, USA; Cat. No. TRCN0000004031) constructed in the pLKO.1-puro plasmid backbone. Briefly, the transfection mixture was prepared by combining 0.1 μg of shRNA plasmid DNA and 0.3 μL of TransIT-LT1 transfection reagent (Mirus Bio LLC, Madison, WI, USA; Cat. No. MIR 2300) in a total volume of 200 μL of Opti-MEM reduced-serum medium. After incubation at room temperature for 20 min, the mixture was added dropwise to the cells. The cells were then incubated for exactly 48 h prior to downstream expression and functional analyses to ensure maximal protein turnover. All transfection experiments were performed in at least three independent biological replicates, with non-targeting shRNA plasmids used as negative controls. The efficiency of UBE2C silencing was subsequently validated at both the mRNA and protein levels via qPCR and Western blot analysis, respectively [11,33].

### 2.15 Immunofluorescence Assay

Human cervical cancer cell lines (HeLa, CC7T/VGH, and CaSki) were seeded onto glass coverslips for immunofluorescence analysis. Cells and tissues were fixed with 4% paraformaldehyde (Thermo Fisher Scientific, Waltham, MA, USA; Cat. No. AAJ19943K2) for 30 min at room temperature, then permeabilized with 0.5% Triton X-100 (Sigma-Aldrich, St. Louis, MO, USA; Cat. No. X100) for 10 min, followed by chilled methanol at −20°C for 10 min. After washing twice with phosphate-buffered saline (PBS), cells were blocked with 10% BSA (Biotools, Taipei, Taiwan; Cat. No. TT-B01) in PBS for 1 h at room temperature to minimize non-specific binding. The samples were then incubated overnight at 4°C with the primary mouse anti-UBE2C antibody (Abnova, Taipei, Taiwan; Cat. No. H00011065-M01) at a dilution of 1:200. Following three 5-min washes with PBS, cells were incubated with a secondary goat anti-mouse Alexa Fluor 488 antibody (Thermo Fisher Scientific, Waltham, MA, USA; Cat. No. A-11001) at a dilution of 1:500 for 1 h at room temperature in the dark. Nuclei were counterstained with DAPI (Thermo Fisher Scientific, Waltham, MA, USA; Cat. No. D1306) at a concentration of 1 μg/mL for 10 min at room temperature, followed by three final washes with PBS to remove excess dye. Fluorescence signals were observed and captured using a Zeiss Axio Observer A1 fluorescence microscope (Carl Zeiss, Oberkochen, Germany). All experiments were performed in at least three independent biological replicates.

### 2.16 Western Blot Analysis

For protein extraction, 1 × 10^6^ cells were lysed in 200 μL RIPA buffer (50 mM Tris-HCl (pH 7.5), 150 mM NaCl, 1 mM EDTA, 1% Nonidet P-40, 0.5% DOC, 0.1% SDS) containing Complete Protease Inhibitor Cocktail (Roche Applied Science, Indianapolis, IN, USA; Cat. No. 11836153001). Cell lysates were centrifuged at 14,000× *g* for 30 min at 4°C, then the supernatant was harvested. Proteins were quantified using the Bio-Rad DC Protein Assay kit (Bio-Rad Laboratories, Hercules, CA, USA; Cat. No. 5000111). Equal amounts of protein (30 μg per lane) were separated by 10% SDS-PAGE and transferred onto PVDF membranes (Millipore, Billerica, MA, USA; Cat. No. IPVH00010). Membranes were blocked with 5% non-fat milk for 1 h at room temperature. Subsequently, membranes were blotted with primary antibodies against UBE2C (Abnova, Taipei, Taiwan; Cat. No. H00011065-M01; 1:1000 dilution), cyclin B1 (Genetex Inc., Irvine, CA, USA; Cat. No. GTX100911; 1:1000 dilution), and anti-α-tubulin (GeneTex Inc, Irvine, CA, USA; Cat. No. GTX628802; 1:1000 dilution) overnight at 4°C. After washing, membranes were incubated with HRP-conjugated secondary antibodies (Goat anti-Mouse IgG, Cat. No. 31430; Thermo Fisher Scientific, Waltham, MA, USA) at a 1:5000 dilution for 1 h at room temperature. Protein bands were detected using an enhanced chemiluminescence (ECL) detection system (Bio-Rad Laboratories, Hercules, CA, USA; Cat. No. 1705060) and visualized with a ChemiDoc Imaging System (Bio-Rad Laboratories).

### 2.17 Prediction of Drug Response Based on UBE2C Expression Using Pharmacogenetics Analysis

To investigate the therapeutic potential of targeting UBE2C, we utilized the Q-omics web application (https://qomics.ai/) to analyze large-scale pharmacogenomics datasets [32]. Drug sensitivity data (IC50 values) were integrated from the Genomics of Drug Sensitivity in Cancer (GDSC, version 2.0) and the Cancer Cell Line Encyclopedia (CCLE), while gene dependency scores were retrieved from the DepMap portal (Public 22Q2). Data integration and normalization were performed as follows: gene expression levels from multiple sources were standardized using z-score transformation to ensure cross-platform comparability. Missing values were handled by excluding cell lines with more than 20% missing data, and no imputation was applied to maintain data integrity. Pearson correlation analysis was employed to assess the relationship between UBE2C expression levels and drug sensitivity (ln(IC50)) across various cancer types. A consensus scoring strategy was applied where candidates were prioritized only if a significant negative correlation (R < −0.40, *p* < 0.05) was consistently observed across both the GDSC and CCLE datasets. This integrated model incorporates multi-omics data, including mutations, gene expression, and patient survival, providing a robust framework for identifying Topotecan as a potential UBE2C-targeted therapeutic agent.

### 2.18 Protein-Protein Docking to Predict UBE2C and CCNB1 Binding Affinity

InterEvDock is a server (http://bioserv.rpbs.univ-paris-diderot.fr/services/InterEvDock2/) for protein-protein docking based on a freed rigid-body docking strategy [30]. The 3D structures of the proteins were downloaded from the PDB database (https://www.rcsb.org/) [34]. Briefly, we input the protein 3D structure files of UBE2C in Protein Data Bank (PDB) format (1I7K or 4YII) and CCNB1 in PDB format (2B9R) in the InterEvDock2 server to analyze the protein-protein docking. The systematic docking server implements three major methods, including FRODOCK2 (rigid-body docking), InterEvScore (IES) (scoring function combining a residue-based statistical potential), and statistically optimized atomic potentials for protein-protein docking (SOAP-PP) (atom-based statistical potential). Based on the input structure, the server runs several steps to propose a selection of 10 most likely models for each score (InterEvScore, SOAP-PP, FRODOCK scores) as well as 10 consensus models and 5 most likely interface residues on each protein. We chose and included the best score for the binding affinity between UBE2C and CCNB1. We used BIOVIA Discovery Studio Visualizer 2025 (v25.1.0.24284) software for predicted protein-protein docking 3D structure visualization.

### 2.19 Molecular Docking to Ensemble UBE2C and Topotecan Binding Affinity

Molecular docking was done by cavity-detection-guided blind docking (CB-DOCK2) server (https://cadd.labshare.cn/cb-dock2/) [35]. CB-DOCK2 is a docking method used for protein-ligand blind docking that predicts the binding sites between protein and ligand and calculates the centers and sizes with a novel protein surface curvature-based cavity detection approach (CurPocket). It works in conjunction with AutoDock Vina (version 1.2.0) for template-independent blind docking, and the pipeline of template-based blind docking employs the BioLip2 database (version of 2025.04.23) as the template database [35]. The 3D structures of the ligand were obtained from the Human Metabolome Database (https://hmdb.ca) [36]. To perform the analyses, the UBE2C protein file in PDB format and the Topotecan ligand file in PDB format were used to input in the docking server, and the default five possible coupling cavities were identified. Among these, the one with the highest binding energy was selected based on the highest Vina value obtained. The proteins and ligands were then visualized with the Licorice and Surface options, respectively. The color of the ligand and the proteins were configured by elements. The protein structure files were viewed using NGL viewer (2.0.0-dev.37) for molecular visualization.

### 2.20 Statistical Analysis

The statistical methods used in this study were based on a previously published protocol [37]. All data are shown as the mean ± S.E.M. Normalization methods, including median and upper-quartile scaling, were used to adjust for variability in gene expression counts. Pearson’s correlation coefficient was applied for association testing, while group differences were assessed using Student’s *t*-test or Fisher’s exact test. One-way ANOVA was performed for comparisons involving more than two groups. All statistical analyses were conducted using GraphPad Prism 9.0 (GraphPad Software Inc., Boston, MA, USA). A *p*-value of less than 0.05 was considered statistically significant.

## 3 Results

### 3.1 UBE2C Expression Landscape and Clinical Significance in Cervical Cancer

**Figure 1:**
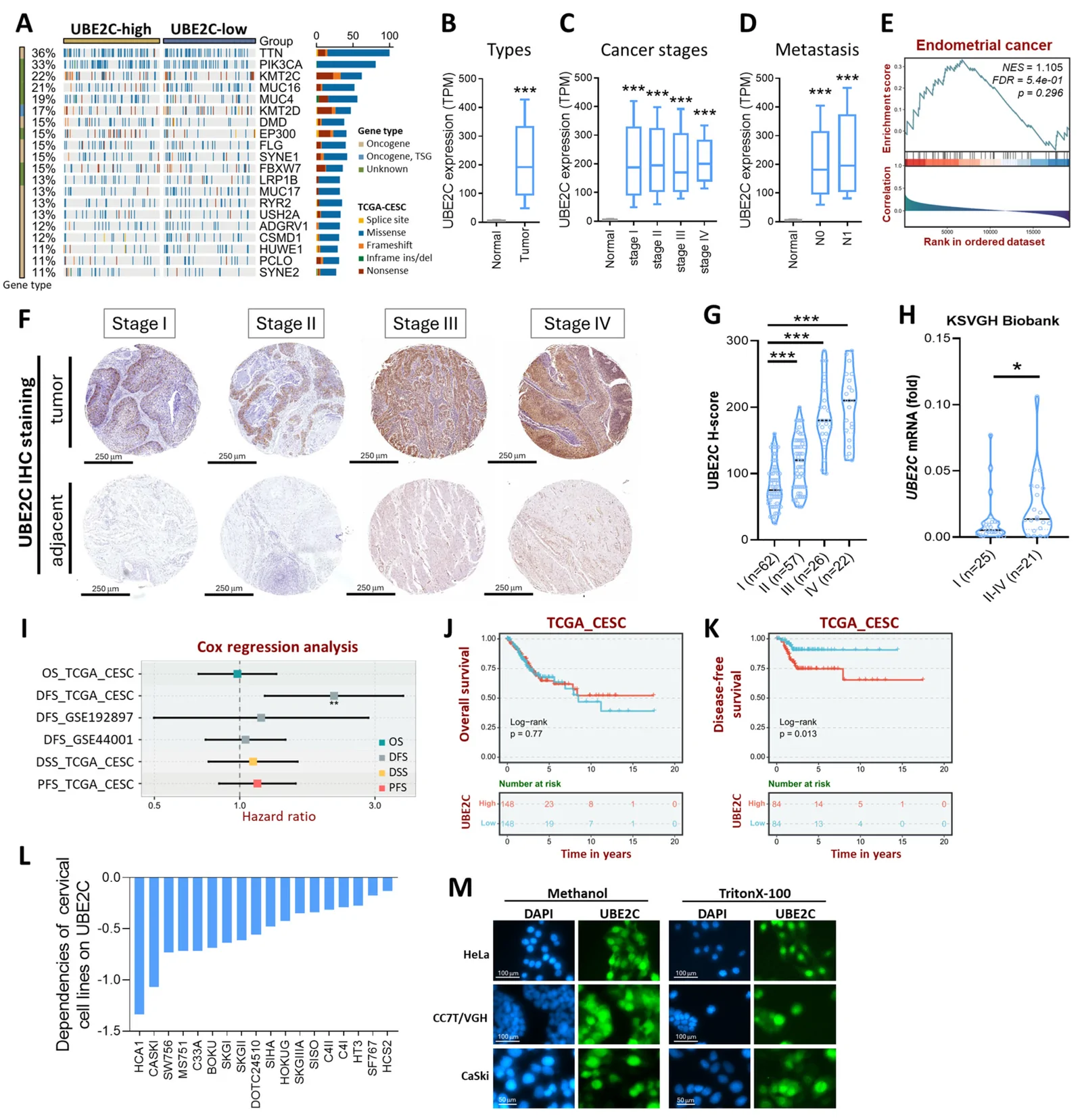
**UBE2C Gene Landscape and Expression in Cervical squamous cell carcinoma and endocervical adenocarcinoma (CESC)**. (**A**) An oncoplot illustrates the mutation frequencies of the top 20 altered genes in patients with high and low UBE2C expression, with the right panel showing mutation types and frequencies. (**B**–**D**) Box plots represent UBE2C expression levels across normal (n = 3) and tumor tissues (n = 305); (**B**) clinical cancer stages I–IV (I: n = 161, II: n = 69, III: n = 46, IV: n = 22); (**C**) and tumor metastasis status (N0: (n = 133) vs. N1 (n = 60)); (**D**) using TCGA database. (**E**) GSEA plot showing the enrichment of endometrial cancer-related pathways in UBE2C-high patients, though the association did not reach statistical significance. (**F**–**H**) Representative IHC images display UBE2C protein levels in tumor tissues across stages compared to adjacent normal tissues (**F**), with corresponding H-score analysis (**G**), quantifying these expression differences. Quantitative PCR validation of UBE2C mRNA expression in Stage I versus Stage II–IV samples from the KSVGH biobank (**H**). (**I**) Forest plot depicting Cox regression analysis of the effect of UBE2C expression levels on the probability of Overall Survival (OS), Disease-Free Survival (DFS), Disease-Specific Survival (DSS), Progression-free survival (PFS) across multiple datasets including TCGA-BRCA, GSE192897 and GSE44001. (**J**,**K**) Kaplan-Meier plot illustrating the probability of OS (**J**) and DFS (**K**) based on UBE2C expression levels in the TCGA cohort, with statistical significance determined by the log-rank test. (**L**) Dependency scores of 18 cervical cancer cell lines on UBE2C are presented based on CRISPR-Cas9 screening data from the Achilles project. (**M**) Representative immunofluorescence images show the intracellular localization and distribution of UBE2C shown in green in HeLa, CC7T/VGH, and CaSki cells, with nuclei counterstained with DAPI shown in blue. Statistical significance is indicated as follows: *p < 0.05, **p < 0.01, ***p < 0.001.

To characterize the role of UBE2C in cervical squamous cell carcinoma and endocervical adenocarcinoma (CESC), we first integrated genomic and transcriptomic data from The Cancer Genome Atlas (TCGA). The mutational landscape was compared between UBE2C-high and UBE2C-low groups using a waterfall plot of the top 20 most frequently mutated genes (Fig. 1A). We observed that the UBE2C-high group exhibited a higher frequency of mutations in genes such as *TTN*, *PIK3CA*, and *KMT2C*, while showing a lower mutation frequency in *HUWE1* compared to the low-expression group. Analysis of TCGA expression profiles revealed that UBE2C was significantly upregulated in tumor tissues compared to normal controls (Fig. 1B). Furthermore, UBE2C levels were found to increase progressively with advanced clinical stages (Fig. 1C) and were significantly higher in patients with lymph node metastasis (N1) compared to those without (N0) (Fig. 1D). While Gene Set Enrichment Analysis (GSEA) showed a trend of enrichment in endometrial cancer-related pathways, it did not reach statistical significance (Fig. 1E). To validate these findings in a clinical cohort, we performed immunohistochemistry (IHC) on 167 CESC samples (Fig. 1F). UBE2C protein expression was markedly higher in tumor tissues than in adjacent normal tissues, with H-scores significantly increasing from Stage I through Stage IV (Fig. 1G). This was further corroborated by qPCR analysis of 25 patient samples from the KSVGH biobank, which demonstrated a significant upregulation of UBE2C mRNA in advanced stages (II–IV) compared to Stage I (Fig. 1H).

Furthermore, we then employed Cox regression hazard ratios and forest plots to produce 95% confidence intervals for various survival outcomes across multiple independent datasets (Fig. 1I). To assess the prognostic relevance of UBE2C on OS (Fig. 1J) and DFS (Fig. 1K) in CESC patients, we performed Kaplan-Meier analysis together with log-rank tests. These analyses demonstrated that high UBE2C expression was significantly associated with poor prognosis and DFS outcomes compared to low UBE2C expression in CESC patients, reinforcing its potential as a prognostic biomarker (Fig. 1J,K). To broaden the evaluation, we also examined data from the Achilles project, which applies CRISPR Cas9–based knockout screening to identify genes required for cancer cell viability. This comprehensive analysis of the dependence of 18 cervical cancer cell lines on UBE2C ensued, with the UBE2C dependence of these cells presented in a ranked fashion based on increased UBE2C dependence (Fig. 1L). In addition, to ascertain the UBE2C localization and distribution in cervical cancer cells, we observed using immunofluorescence staining, encompassing UBE2C and DAPI in three cervical cancer cell lines (HeLa, CC7T/VGH and CaSki). As depicted in Fig. 1M, the intracellular localization and distribution of UBE2C is higher in these cervical cancer cells. Taken together, these findings suggest that high UBE2C expression is strongly linked with tumor survival capacity and may represent both a prognostic biomarker and a potential therapeutic target in cervical cancer.

### 3.2 UBE2C Modulates the Immunosuppressive Microenvironment and Macrophage Recruitment

**Figure 2:**
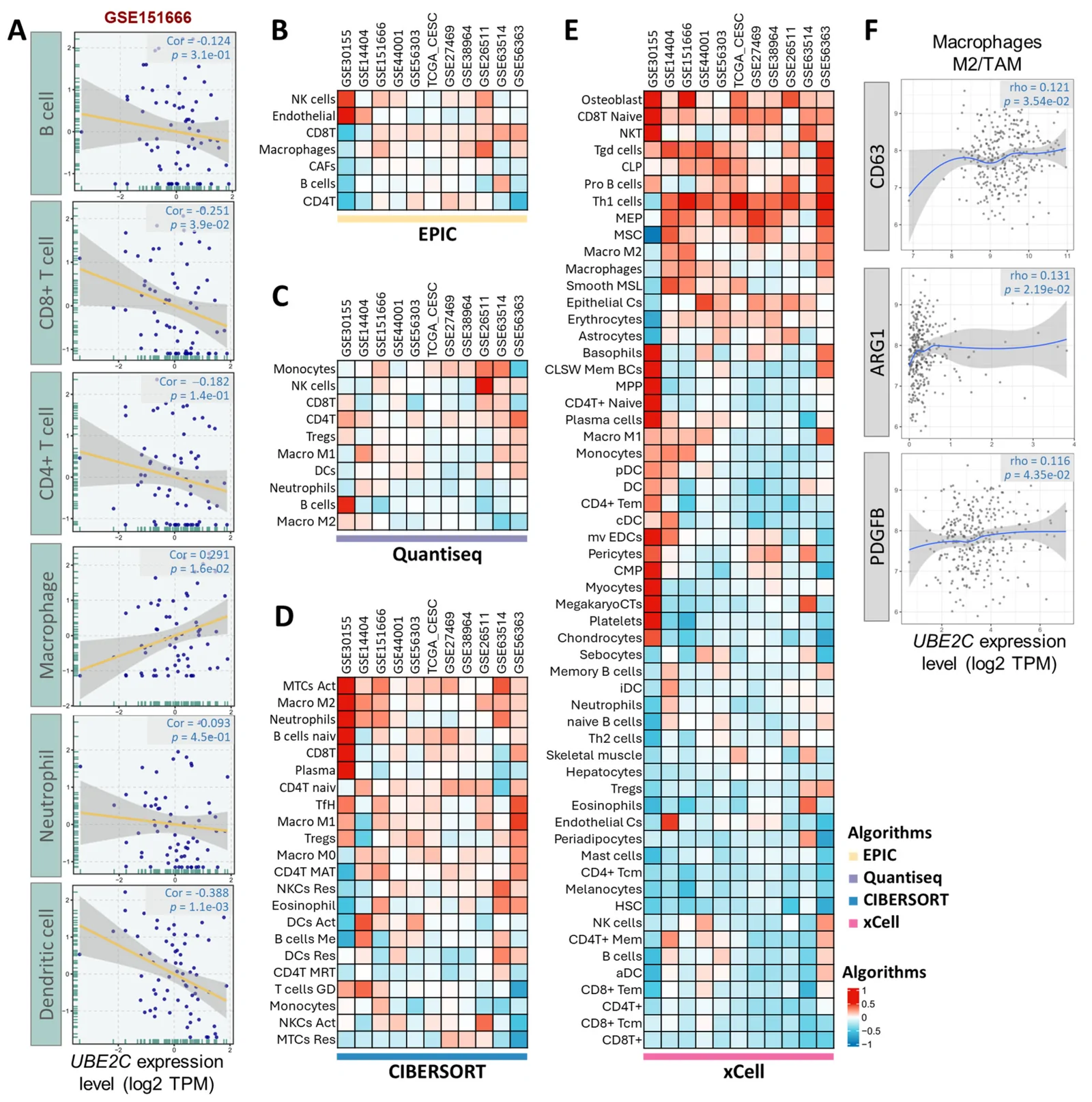
**Association between UBE2C Expression with Immune Infiltration in CESC.** (**A**) Scatter plots showing the correlation between UBE2C expression and the infiltration levels of B cells, CD8^+^ T cells, CD4^+^ T cells, macrophages, neutrophils, and dendritic cells in the GSE151666 dataset. (**B**) Heatmaps illustrating correlation coefficients between UBE2C expression and various immune cell types across 11 independent cervical cancer datasets, calculated using four different algorithms: EPIC (**B**), Quantiseq (**C**), CIBERSORT (**D**), and xCell (**E**). Red indicates positive correlation, while blue indicates negative correlation. (**F**) Correlation analysis between UBE2C expression and specific markers associated with M2 macrophages/TAMs, including CD63, ARG1, and PDGFB.

To characterize the relationship between UBE2C and the cervical cancer immune landscape, we analyzed immune cell infiltration patterns using the GSE151666 dataset. Correlation analysis revealed that UBE2C expression is significantly and positively associated with macrophage infiltration, while exhibiting negative correlations with cytotoxic populations such as CD8^+^ T cells and dendritic cells (Fig. 2A). This infiltrative pattern was robustly validated across 11 independent datasets using four distinct deconvolution algorithms—EPIC (Fig. 2B), Quantiseq (Fig. 2C), CIBERSORT (Fig. 2D), and xCell (Fig. 2E)—all of which consistently confirmed a strong positive correlation between UBE2C levels and macrophage populations (Fig. 2B–E).

To investigate the underlying mechanisms driving this recruitment, we examined the association between UBE2C and the secretable cytokine milieu. UBE2C expression showed a notable positive correlation with growth factor VEGFB in CESC (Supplementary Fig. S1A,B). Furthermore, analysis of Gene Ontology (GO) functional scores demonstrated that the UBE2C-high group exhibited significant shifts in pathways related to cytokine production, inflammatory responses, and TGF-β production compared to the UBE2C-low group in CESC (Supplementary Fig. S1C). Furthermore, transcriptomic profiling revealed that while high sgUBE2C efficacy enhanced cell migration via the VEGF signaling pathway, it significantly reduced the positive regulation of macrophage migration and elevated the negative regulation of chemotaxis (Supplementary Fig. S1D), providing a mechanistic link between UBE2C levels and macrophage infiltration. At the molecular level, UBE2C expression displayed robust positive correlations with specific markers of M2-type TAMs, including CD63, ARG1, and PDGFB (Fig. 2F). To experimentally validate these associations, we performed qPCR analysis following shRNA-mediated knockdown of UBE2C. The silencing of UBE2C led to significant alterations in the mRNA levels of key inflammatory mediators, including a prominent upregulation of IL10 (Supplementary Fig. S1E). Collectively, these findings from both Fig. 2 and Supplementary Fig. S1 demonstrate that UBE2C fosters an immunosuppressive microenvironment by modulating cytokine signaling and facilitating the spatial recruitment of M2-polarized macrophages in CESC.

### 3.3 Spatial Transcriptomic Characterization of UBE2C and the Immune Microenvironment

**Figure 3:**
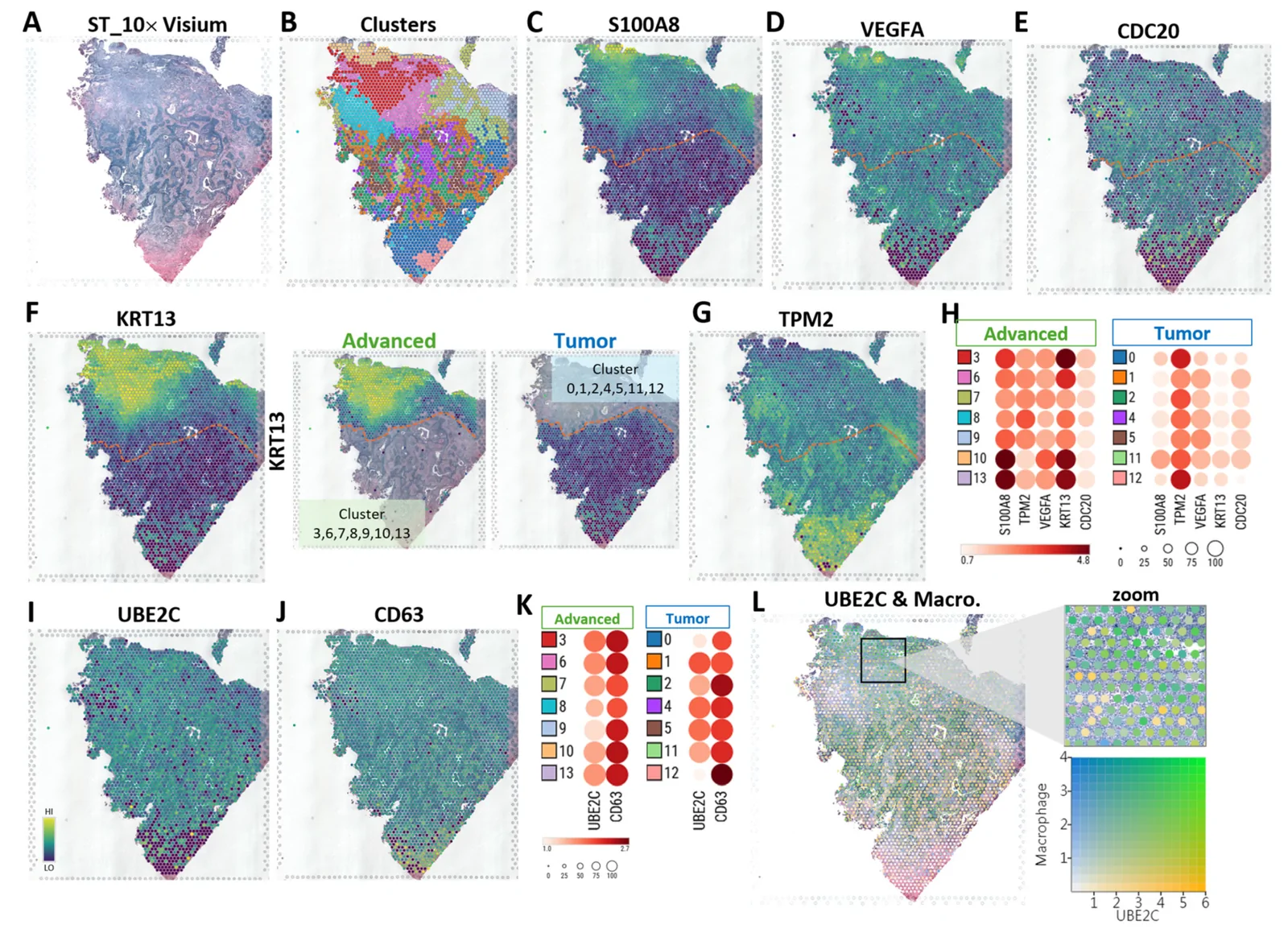
**Spatial transcriptomic characterization and regional identification in CESC patients.** (**A**) H&E-stained section of a CESC tumor biopsy processed via 10× Visium spatial transcriptomics. (**B**) Unsupervised clustering identifying 14 distinct clusters (0–13) based on global gene expression. (**C**–**G**) Spatial expression maps of pro-tumor markers S100A8 (**C**), VEGFA (**D**), and CDC20 (**E**). (**F**) Spatial expression of KRT13 and the corresponding partitioning of clusters into “Advanced” and “Tumor” regions based on biomarker enrichment patterns. (**G**) Spatial expression map of the tumor-suppressive marker TPM2. (**H**) Dot plot illustrating expression frequency and average intensity of five key biomarkers across individual clusters within the “Advanced” and “Tumor” regions. (**I**) Spatial expression map of the cell cycle-related gene *UBE2C*. (**J**) Spatial expression map of the macrophage marker *CD63*. (**K**) Dot plot showing the expression levels and frequencies of *UBE2C* and *CD63* across all clusters. (**L**) Bivariate colocalization analysis (with zoom-in view) evaluating the spatial overlap between *UBE2C* expression and macrophage signatures.

To further investigate the intratumoral heterogeneity of cervical cancer, we utilized 10x Visium spatial transcriptomics to map transcriptomic signatures onto H&E-stained histological sections of CESC tumor tissue (Fig. 3A). Unsupervised clustering of the capture spots identified 14 distinct spatial clusters, revealing a highly organized architectural landscape (Fig. 3B). Spatial feature plots demonstrated clear regional gradients of established biomarkers. Specifically, oncogenic or pro-tumor markers, including *S100A8* (Fig. 3C), *VEGFA* (Fig. 3D), *CDC20* (Fig. 3E), and *KRT13* (Fig. 3F), were predominantly enriched in the upper tissue regions. Conversely, the tumor-suppressive marker *TPM2* (Fig. 3G) exhibited higher expression levels in the lower regions.

Based on these divergent expression patterns, we categorized the identified clusters into two major functional domains (Fig. 3F): the “Advanced” region, characterized by high *KRT13* expression (comprising clusters 3, 6, 7, 8, 9, 10, and 13), and the “Tumor” region, characterized by *TPM2* enrichment (comprising clusters 0, 1, 2, 4, 5, 11, and 12). The “Advanced” region closely aligns with areas showing heightened oncogenic signaling activity. Dot plot analysis corroborated these findings, demonstrating that markers associated with tumor progression and the cell cycle, such as *CDC20*, were significantly more abundant in the “Advanced” region than in the “Tumor” region (Fig. 3H).

We next focused on the spatial distribution of *UBE2C* and the macrophage-related marker *CD63*. While *UBE2C* was broadly distributed across the tumor sections, it displayed localized hotspots of high intensity within specific clusters (Fig. 3I). In contrast, *CD63* expression was more selectively concentrated within the “Advanced” domain, with Cluster 10 exhibiting the most prominent enrichment, suggesting a localized niche for macrophage activity (Fig. 3J,K). Bivariate colocalization analysis further visualized the spatial relationship between *UBE2C* expression and macrophage infiltration (Fig. 3L). The analysis highlighted a strong spatial concordance between *UBE2C*-high cells and macrophage populations, particularly within the malignant clusters of the “Advanced” region. These spatial insights suggest that *UBE2C* expression parallels regional tumor malignancy and is closely associated with the presence of macrophages within the CESC microenvironment.

### 3.4 Single-Cell Transcriptomic Analysis Identifies SPI1 as a Potential Regulator of UBE2C in Macrophages

**Figure 4:**
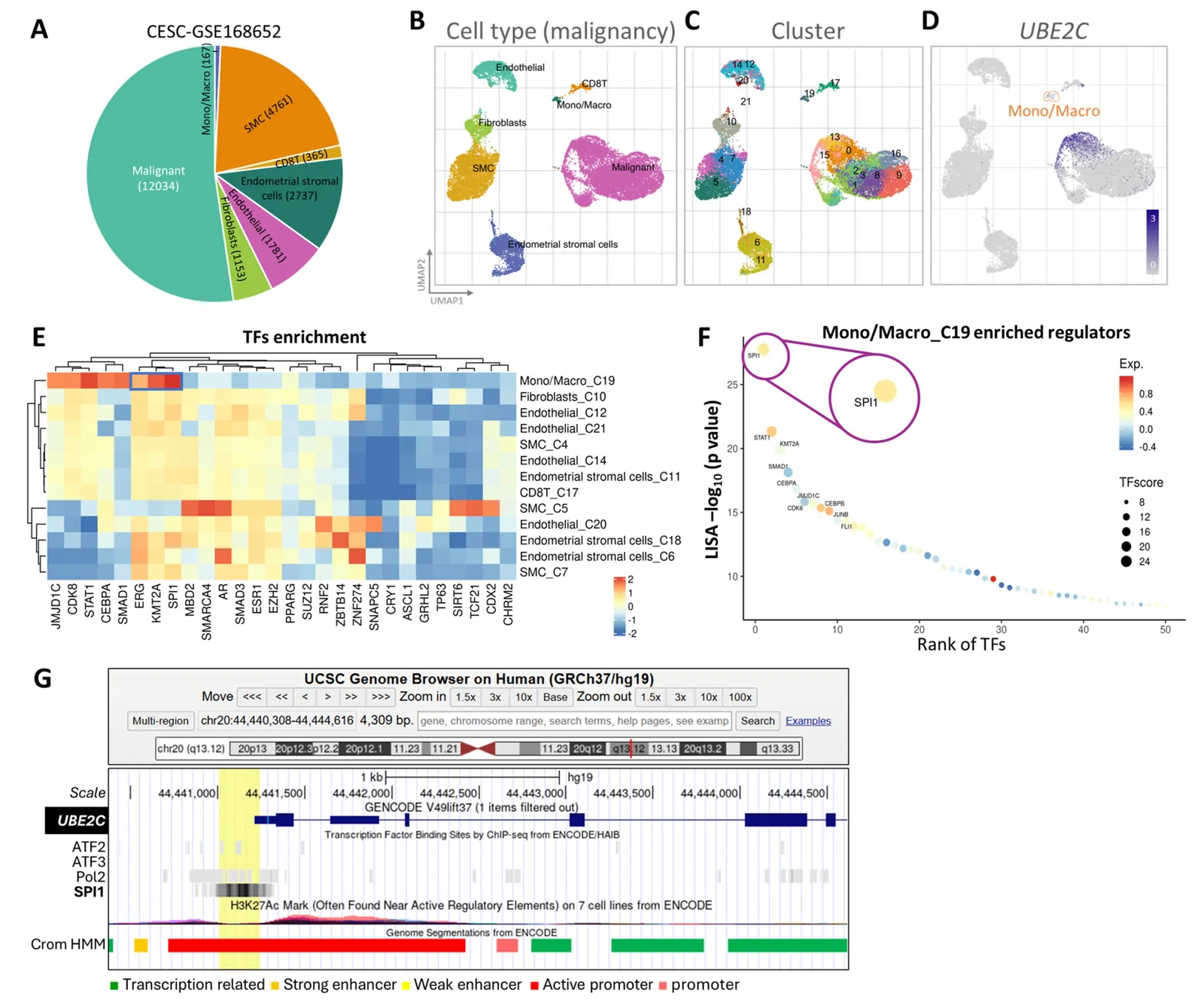
**Single-cell transcriptomic evaluation and transcriptional regulation of UBE2C.** (**A**) Pie chart illustrating the relative proportions of major cell populations in the CESC-GSE168652 dataset, including Malignant cells, Smooth Muscle Cells (SMC), and Endometrial stromal cells. (**B**) UMAP plot displaying single cells color-coded according to their identified cell types and malignancy status. (**C**) UMAP plot showing the distribution of 22 distinct clusters (labeled 0–21) identified through unsupervised clustering analysis. (**D**) UMAP plot highlighting cluster-specific expression of UBE2C, with high expression levels concentrated in the Mono/Macro population. (**E**) Heatmap showing transcription factor (TF) enrichment levels for UBE2C and cell type markers across selected single-cell clusters. (**F**) Landscape In Silico deletion Analysis (LISA) rank of transcription factors identifying top enriched regulators for the Mono/Macro_C19 cluster, with SPI1 showing significant enrichment. (**G**) UCSC Genome Browser visualization (GRCh37/hg19) of the UBE2C locus, illustrating integrated ENCODE regulatory tracks including H3K27Ac marks and potential SPI1 binding sites near the promoter region.

To characterize UBE2C expression at single-cell resolution and investigate the heterogeneity of the CESC microenvironment, we analyzed a scRNA-seq dataset (GSE168652) comprising malignant cells and various stromal and immune populations (Fig. 4A). After quality control and batch effect correction, UMAP visualization resolved twelve distinct clusters derived from seven annotated cell types, including malignant, endothelial, smooth muscle, and monocyte/macrophage lineages (Fig. 4B,C). Inspection of UBE2C expression across this cellular landscape revealed that its relative transcript abundance was most concentrated within the monocyte/macrophage cluster in this dataset (Fig. 4D), a pattern we interpret in light of single-cell technical factors and a context-dependent myeloid pool of UBE2C.

To identify potential upstream regulators of UBE2C in these myeloid cells, we performed transcription factor (TF) enrichment analysis across the identified clusters. Heatmap analysis of TF activity identified SPI1 as one of the most prominent regulators enriched in the macrophage-dominant populations (Fig. 4E). Further analysis using the Landscape In Silico deletion Analysis (LISA) framework confirmed that SPI1 ranked as a top regulator for Cluster 19 (C19), which exhibits a strong monocyte/macrophage signature (Fig. 4F). To provide genomic evidence for this regulatory link, we examined the UBE2C gene locus using the UCSC Genome Browser. The integrated ChIP-seq data demonstrated clear SPI1 binding peaks within the UBE2C promoter region, overlapping with active regulatory elements such as H3K27Ac marks and promoter-associated segments (Fig. 4G). These findings suggest that UBE2C expression in TAMs may be directly regulated by the SPI1 transcriptional program, potentially linking macrophage development and function with UBE2C-mediated processes in the cervical cancer microenvironment.

### 3.5 UBE2C Potentially Regulates DNA Replication and Cell Cycle Function

**Figure 5:**
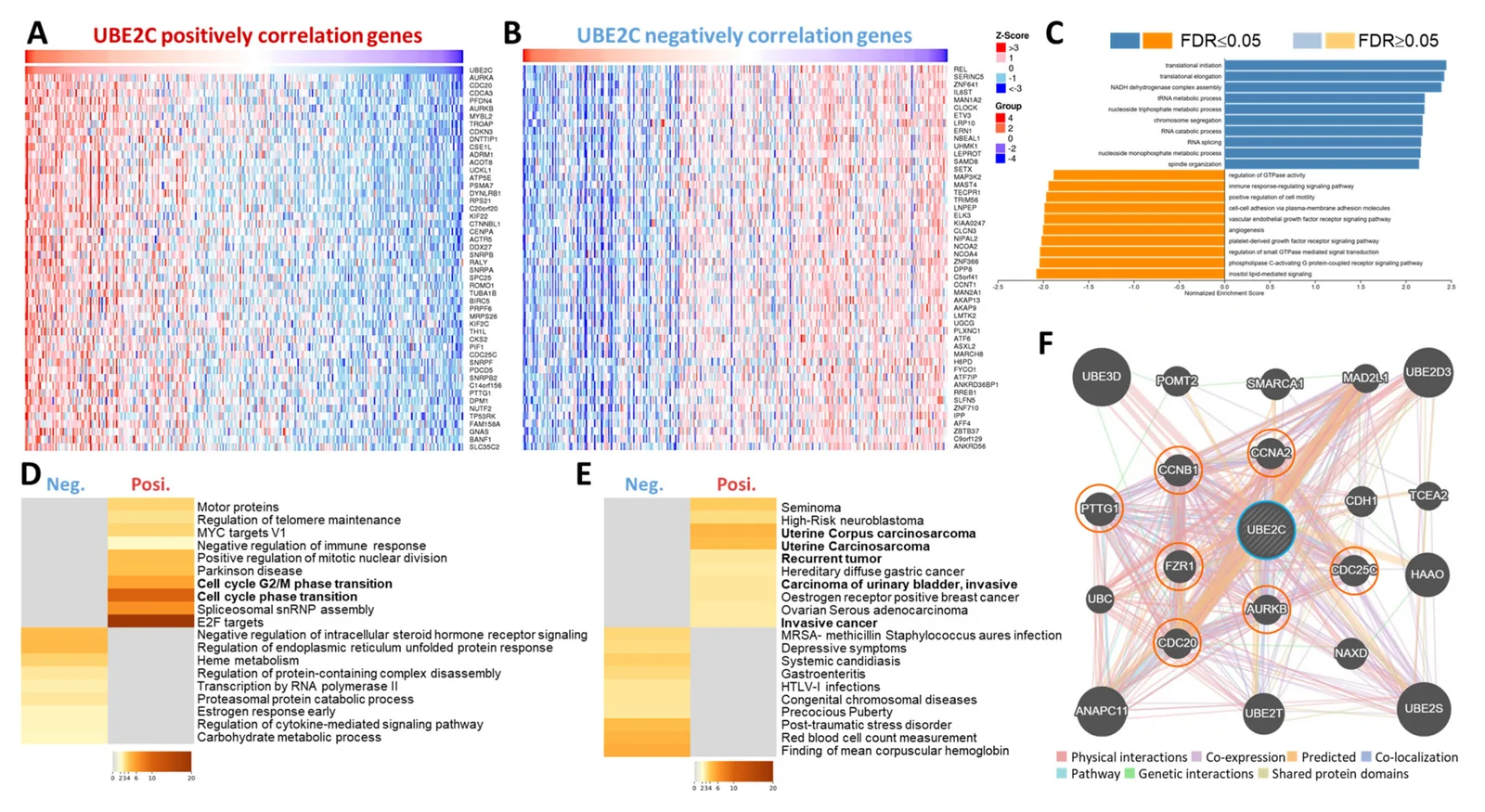
**Investigating the Role of *UBE2C* in Biological Processes.** (**A**) Heatmap displaying the top 50 genes significantly positively correlated with UBE2C expression, analyzed using the LinkedOmics database. (**B**) Heatmap displaying the top 50 genes significantly negatively correlated with UBE2C expression, analyzed using the LinkedOmics database. (**C**) Bar chart illustrating functional enrichment analysis of UBE2C-associated genes in CESC. Blue bars indicate pathways with a False Discovery Rate (FDR) ≤ 0.05, while orange bars represent FDR ≥ 0.05. (**D**) Metascape enrichment analysis showing biological processes and pathways associated with genes positively and negatively correlated with UBE2C expression. (**E**) Enrichment analysis of diseases involving UBE2C-associated genes, conducted using DisGeNET terms within the Metascape database. (**F**) An interactive functional protein-protein interaction (PPI) network of UBE2C and its related genes, constructed using the GeneMANIA database. Different colored edges represent physical interactions, co-expression, predicted associations, and shared protein domains.

To explore the relationship between UBE2C expression and gene signatures in cervical cancer, we conducted a correlation analysis, categorizing the results into positive (Fig. 5A) and negative (Fig. 5B) regulatory genes (Fig. 5A,B). Enrichment analysis indicated that genes positively correlated with UBE2C were associated with translational initiation, translational elongation, NADH dehydrogenase complex assembly, chromosome segregation, and spindle organization. Conversely, negatively correlated genes were linked to inositol lipid-mediated signaling and regulation of small GTPase-mediated signal transduction. angiogenesis, cell-cell adhesion via plasma membrane adhesion molecule, immune-response regulating signaling pathway, and positive regulation of cell motility (Fig. 5C). Moreover, the enrichment analysis revealed that the UBE2C-positively correlated genes were associated with cell cycle phase transition and cell cycle G2/M phase transition (Fig. 5D). Fig. 5E displays the enrichment analysis of diseases particularly cancers such as uterine corpus carcinosarcoma, uterine carcinosarcoma, and carcinoma of urinary bladder were associated with UBE2C-positively correlated genes. A comprehensive analysis revealed that UBE2C engages in physical interactions, co-expression, and co-localization with various cell cycle-related markers (Fig. 5F). Taken together, these findings highlight that UBE2C plays a pivotal role in regulating Cell cycle and DNA replication processes in cervical cancer progression.

### 3.6 UBE2C Promotes Cell Proliferation by Regulating Cell Cycle and DNA Replication Machinery

**Figure 6:**
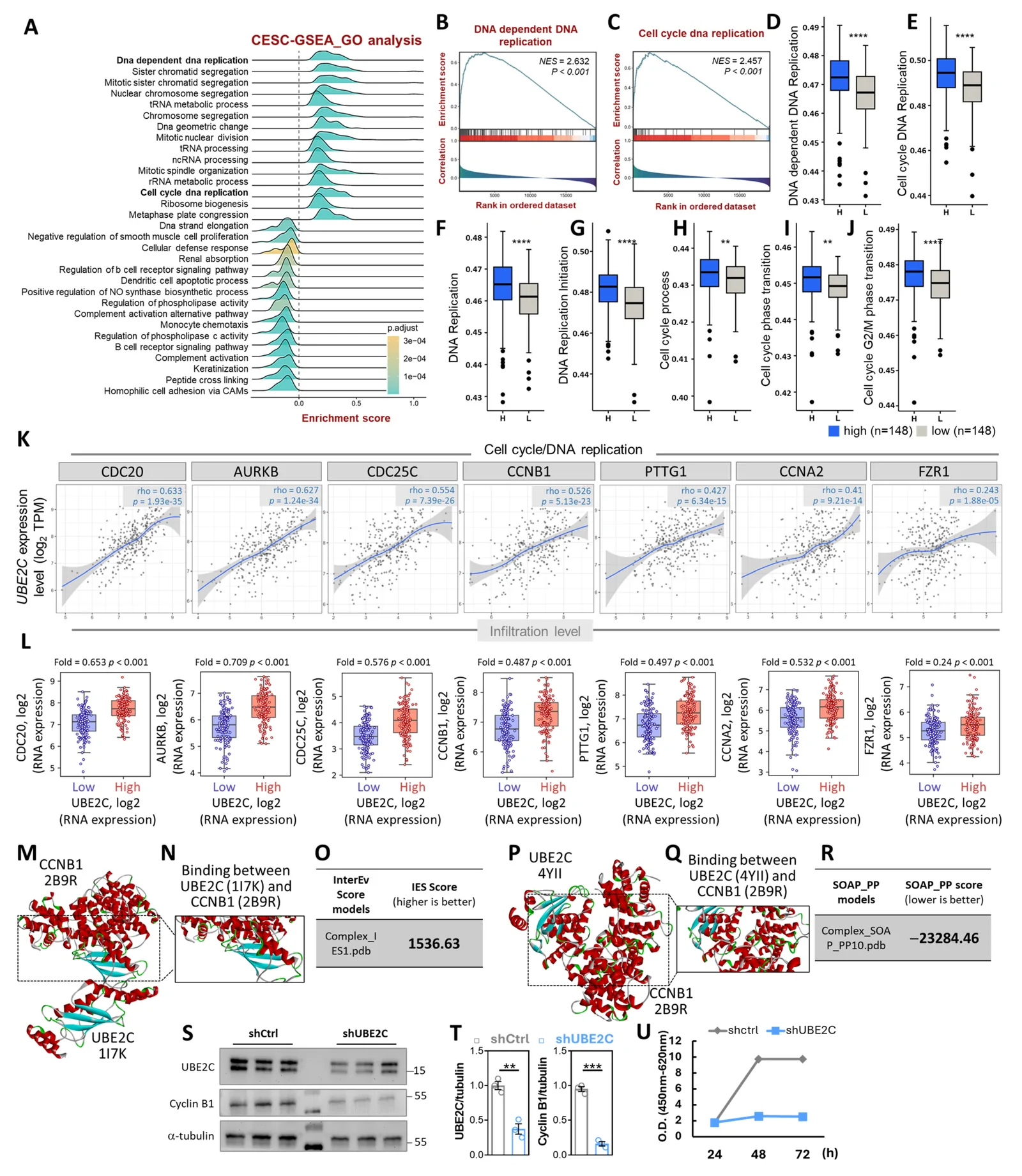
**UBE2C regulates the cell cycle and promotes cell proliferation through interaction with CCNB1.** (**A**) GSEA ridge plot displaying the top 20 Gene Ontology (GO) terms associated with UBE2C expression in CESC, analyzed using the Biomarker Exploration for Solid Tumors (BEST) database. (**B**,**C**) GSEA enrichment plots demonstrating that high UBE2C expression is significantly correlated with “DNA dependent DNA replication” (**B**) and “Cell cycle DNA replication” (**C**) signatures. (**D**–**J**) Boxplots comparing various functional signatures—including DNA dependent DNA replication (**D**), Cell cycle DNA replication (**E**), DNA replication (**F**), DNA replication initiation (**G**), Cell cycle process (**H**), Cell cycle phase transition (**I**), Cell cycle G2/M phase transition (**J**)—between UBE2C-high and UBE2C-low groups. Statistical significance is indicated by asterisks. (**K**) Correlation analysis between UBE2C expression and key cell cycle/DNA replication markers (including CDC20, AURKB, CDC25C, CCNB1, PTTG1, CCNA2, and FZR1) using TIMER3 and Q-omics databases. (**L**) Comparison of the mRNA expression levels of the markers mentioned in (**K**) between patients with low and high UBE2C expression. (**M**–**O**) Molecular docking and protein-protein interaction (PPI) modeling between UBE2C (PDB: 1I7K) and CCNB1 (PDB: 2B9R) also known as Cyclin B1. (**M**) Panels show the predicted docking complexes, (**N**) specific binding interfaces, and (**O**) interaction scores using InterEvScore (IES) metrics. (**P**–**R**) Molecular docking and protein-protein interaction (PPI) modeling between UBE2C (PDB: 4YII) and CCNB1 (PDB: 2B9R). (**P**) Panels show the predicted docking complexes, (**Q**) specific binding interfaces, and (**R**) interaction scores using statistically optimized atomic potentials for protein-protein docking (SOAP-PP) metrics. (**S**) Representative Western blot analysis showing the knockdown efficiency of UBE2C (shUBE2C) and its subsequent effect on Cyclin B1 protein levels. α-tubulin was used as a loading control. (**T**) Quantitative analysis of UBE2C and Cyclin B1 protein levels from Western blot experiments, normalized to tubulin. (**U**) Cell proliferation curve (O.D. 450nm–620nm) over a 72-h period, comparing cell growth between the control (shCtrl) and UBE2C knockdown (shUBE2C) groups. **p < 0.01, ***p < 0.001, ****p < 0.0001.

To explore the biological functions of UBE2C in cervical cancer, we performed GSEA based on Gene Ontology (GO) terms. The analysis revealed that UBE2C expression is predominantly associated with pathways involving DNA-dependent DNA replication and cell cycle DNA replication (Fig. 6A). Specifically, the enrichment plots confirmed a significant positive correlation between UBE2C levels and gene sets related to DNA replication and the cell cycle (Fig. 6B,C). Patients in the UBE2C-high group exhibited significantly higher scores for DNA-dependent DNA replication (Fig. 6D), Cell cycle DNA replication (Fig. 6E), DNA replication (Fig. 6F), DNA replication initiation (Fig. 6G), Cell cycle process (Fig. 6H), Cell cycle phase transition (Fig. 6I), Cell cycle G2/M phase transition (Fig. 6J), compared to the UBE2C-low group (Fig. 6D–J). We further analyzed the correlation between UBE2C and key cell cycle regulators. Transcriptomic data showed that UBE2C expression is robustly and positively associated with several core cell cycle genes, including *CDC20*, *AURKB*, *CDC25C*, *CCNB1*, *PTTG1*, *CCNA2*, and *FZR1* (Fig. 6K,L).

To investigate the potential molecular interaction between UBE2C and its correlated targets, we employed protein-protein docking models. The structural analysis indicated a potential binding affinity between UBE2C (PDB: 1I7K/4YII) and CCNB1 (Cyclin B1, PDB: 2B9R), as evidenced by favorable interaction scores in both InterEvScore and statistically optimized atomic potentials for protein-protein docking (SOAP-PP) models (Fig. 6M–R). Finally, we performed functional validation using shRNA-mediated knockdown of UBE2C in cervical cancer cells. Western blot analysis demonstrated that the silencing of UBE2C led to a significant reduction in CCNB1 protein levels (Fig. 6S,T). Consistent with these molecular changes, the cell proliferation assay showed that UBE2C knockdown markedly suppressed the growth rate of cervical cancer cells over time (Fig. 6U). Collectively, these findings suggest that UBE2C drives cervical cancer progression by regulating the stability or expression of key cell cycle regulators like CCNB1, thereby promoting cell cycle, DNA replication, and cell proliferation.

### 3.7 Multi-Omics Validation of Topotecan as a UBE2C-Targeting Therapeutic Strategy

**Figure 7:**
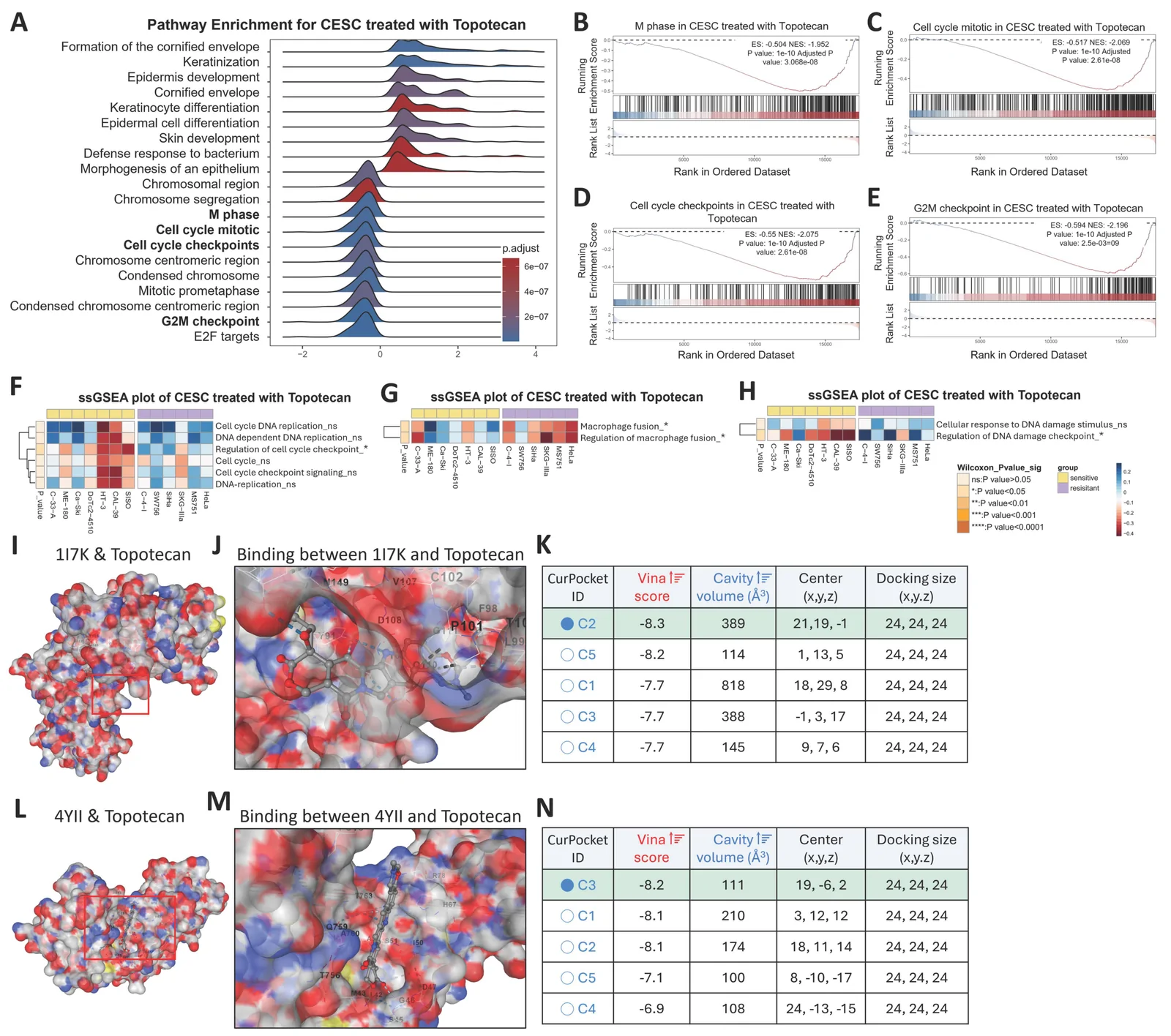
**Pharmacogenomic profiling and molecular docking identify Topotecan as a therapeutic lead for UBE2C-high cervical cancer.** (**A**) Ridge plot showing the top 20 significantly enriched biological pathways in CESC treated with Topotecan, highlighting alterations in cell cycle-related processes and DNA replication. (**B**–**E**) GSEA plots illustrating the enrichment of specific gene sets—including M phase (**B**), cell cycle mitotic (**C**), cell cycle checkpoints (**D**), and G2/M checkpoint (**E**)—in response to Topotecan treatment. (**F**–**H**) ssGSEA heatmaps evaluating the enrichment levels of pathways related to cell cycle/DNA replication (**F**), macrophage fusion (**G**), and DNA damage response (**H**) across Topotecan-sensitive and resistant cervical cancer cell lines. (**I**–**N**) Protein-Ligand binding affinity of UBE2C and Topotecan were identified and visualized using molecular docking. (**I**) 3D visual representation of the molecular docking structure between the UBE2C (1I7K) protein and the Topotecan ligand. (**J**) Detailed view of the binding pocket and intermolecular interactions between Topotecan and specific amino acid residues of the 1I7K protein. (**K**) Data table summarizing the docking parameters for 1I7K, including CurPocket ID, Vina score, cavity volume, and coordinates of the docking center. (**L**) 3D visual representation of the molecular docking structure between the UBE2C (4YII) protein and the Topotecan ligand. (**M**) Detailed view of the binding interface and molecular interactions between Topotecan and the 4YII protein pocket. (**N**) Data table listing the docking results for 4YII, showing Vina scores for different potential binding sites (C1–C5) and their respective spatial configurations.

To investigate the therapeutic potential and mechanism of Topotecan in cervical cancer, we analyzed transcriptomic changes in CESC cells treated with Topotecan. Gene Set Enrichment Analysis (GSEA) plots revealed that Topotecan treatment is predominantly associated with the down-regulation of pathways essential for tumor progression, including M phase, cell cycle mitotic, cell cycle checkpoints, and the G2M checkpoint (Fig. 7A–E). Further analysis using ssGSEA across various CESC cell lines identified distinct signatures associated with Topotecan sensitivity and resistance, particularly in pathways related to Regulation of cell cycle checkpoints, macrophage regulation, and DNA damage checkpoints (Fig. 7F–H).

To further validate these findings, we examined independent pharmacogenomic datasets across multiple cancer types. In uterine corpus endometrial carcinoma (UCEC), Topotecan similarly suppressed mitotic progression and G2/M-related pathways, while UBE2C expression was significantly associated with drug response sensitivity (Supplementary Fig. S2A–K). In ovarian cancer cell lines, Topotecan induced concentration- and time-dependent downregulation of multiple cell-cycle regulators, including UBE2C, CCNB1, CDC25C, and CDC20 (Supplementary Fig. S3A–P). In urinary tract cancer models, transcriptomic analyses demonstrated that Topotecan significantly altered genes involved in DNA replication, RNA splicing, and proliferative signaling pathways (Supplementary Fig. S4A–D). Furthermore, genetic perturbation analysis revealed that depletion of UBE2 family members, particularly UBE2K, was significantly associated with increased sensitivity to Topotecan treatment (Supplementary Fig. S5A–G). Consistently, Topotecan treatment also reversed UBE2C-associated transcriptional signatures and preferentially inhibited cell lines enriched for G2 phase programs, further linking UBE2C dependency to therapeutic response (Supplementary Fig. S6A–F).

Based on the observed inhibition of cell cycle pathways, we explored whether Topotecan could directly interact with UBE2C, a key regulator of the cell cycle identified in our previous analyses. Molecular docking was performed using the protein structures of UBE2C (PDB: 1I7K and 4YII). The simulation identified specific binding pockets where Topotecan could stably interact with UBE2C, as indicated by favorable Vina scores and identified binding cavities (Fig. 7I–N). These structural models suggest a potential direct inhibitory effect of Topotecan on UBE2C activity. Overall, these results predict that UBE2C can be used as a promising target for the development of topotecan treatment strategies in cervical cancer.

## 4 Discussion

CESC is characterized by profound dysregulation of cell cycle–related pathways, reflecting its highly proliferative and genomically unstable nature. Integrated genomic analyses from TCGA have demonstrated that genes involved in mitotic progression and chromosomal segregation are among the most consistently upregulated molecular programs in cervical cancer, underscoring the central role of aberrant cell cycle control in disease pathogenesis [38]. In this context, UBE2C, a core component of the anaphase-promoting complex/cyclosome (APC/C) machinery, has emerged as a recurrently overexpressed gene across multiple cancer types, including gynecological malignancies. Recent pan-cancer analyses based on TCGA and other large transcriptomic datasets have confirmed that UBE2C expression is significantly elevated in a broad spectrum of solid tumors and is closely associated with proliferative signatures and unfavorable clinical outcomes [39].

The results of this study demonstrate that UBE2C is a critical oncogenic driver in cervical squamous cell carcinoma and plays a multifaceted role in modulating both the cell cycle and the immunological microenvironment. Clinical analysis of multiple cohorts indicates that UBE2C expression is significantly elevated in tumor tissues and serves as an independent predictor of poor disease-free survival [1,40]. These findings are consistent with the known function of UBE2C as a key regulator of the ubiquitin proteasome system, where it facilitates the degradation of mitotic proteins to promote uncontrolled cellular proliferation [5,7]. The oncogenic role of UBE2C is primarily attributed to its function in cell cycle regulation. UBE2C catalyzes ubiquitin transfer to APC/C substrates, thereby promoting the timely degradation of key mitotic regulators such as cyclin B1 [41]. Tight regulation of UBE2C expression is essential for maintaining genomic stability, whereas its overexpression leads to premature or unscheduled substrate degradation, resulting in chromosomal instability (CIN) and aneuploidy [42]. Our dependency screens further confirm that cervical cancer cells are highly reliant on UBE2C for maintaining viability, reinforcing its potential as a therapeutic target.

CESC is recognized as an immunologically active tumor type, characterized by substantial infiltration of lymphoid and myeloid cells [43]. Particularly, TAMs represent a dominant immune population in cervical cancer and have been implicated in tumor progression, angiogenesis, and immune suppression [17]. While UBE2C expression is predominantly confined to malignant epithelial cells, our data reveals a robust association between UBE2C-high tumor regions and macrophage-enriched microenvironments, suggesting a potential link between tumor cell proliferative status and myeloid cell recruitment or retention.

Macrophages exhibit remarkable functional plasticity and can adopt a spectrum of activation states in response to microenvironmental cues [44]. The classical dichotomy between pro-inflammatory M1-like and immunosuppressive M2-like macrophages provides a useful conceptual framework for understanding TAM biology, although it does not fully capture the complexity of macrophage phenotypes *in vivo* [45,46]. In cervical cancer, increased infiltration of M2-like TAMs has been associated with poor prognosis, enhanced tumor invasion, and resistance to therapy [47,48,49].

It is worth noting that our single-cell analysis localized the highest relative UBE2C transcript abundance to the monocyte/macrophage compartment (Fig. 4D), which may appear to contrast with the tumor-intrinsic role emphasized throughout this study. We interpret this pattern as reflecting both technical and biological considerations rather than a true reassignment of UBE2C to myeloid cells. Technically, droplet-based scRNA-seq is subject to preferential loss of malignant epithelial cells during tissue dissociation, high gene-level dropout, and per-cell library-size normalization, all of which tend to dilute the signal of cell-cycle–restricted transcripts such as UBE2C across a large, predominantly non-cycling malignant cluster while concentrating it within the more compact macrophage cluster; the cellular composition of the specific dataset analyzed (GSE168652) further shapes these relative proportions. Biologically, the macrophage-associated signal is consistent with a context-dependent, SPI1-regulated pool of UBE2C in TAMs (Fig. 4E–G), which may coexist with rather than replace its dominant tumor-cell–autonomous function. Importantly, the tumor-intrinsic role of UBE2C in this study is established independently of the single-cell data, being supported by tumor-restricted immunohistochemical staining across 167 specimens (Fig. 1F,G), immunofluorescence in cervical cancer cell lines (Fig. 1M), CRISPR-Cas9 dependency screens (Fig. 1L), and shRNA knockdown that reduces CCNB1 and suppresses proliferation (Fig. 6S–U). The single-cell distribution should therefore be regarded as a relative, platform- and dataset-dependent observation that complements—rather than contradicts—the tumor-intrinsic and macrophage-associated dimensions of UBE2C biology described here.

Our transcriptomic spatial analyses reveal a robust correlation between UBE2C-high tumor regions and macrophages. Using four independent deconvolution algorithms, we consistently observed that elevated UBE2C levels are associated with increased macrophage infiltration and activation scores across diverse datasets. Notably, this correlation was also supported by immune database analyses, which yielded consistent results. Spatial transcriptomics further delineated the architecture of this interaction, identifying hotspots where UBE2C expression co-localizes with the macrophage marker CD63 in advanced tumor domains [17,50]. Instead, these findings support an indirect relationship, wherein highly proliferative tumor cells with elevated UBE2C may shape the surrounding immune microenvironment through altered cytokine, chemokine, or metabolic signaling, thereby establishing supportive immune niches.

Such tumor-immune crosstalk has been reported in other cancer types, where rapidly cycling tumor cells influence macrophage recruitment and polarization through the secretion of factors such as CSF-1, CCL2, and VEGF [51,52]. Although direct evidence linking UBE2C activity to macrophage polarization is currently lacking, our data suggest that UBE2C-associated proliferative states may serve as a surrogate marker for tumor regions that are permissive to macrophage accumulation and functional skewing. The observed association between UBE2C overexpression and macrophage-enriched TMEs has several important biological and clinical implications [53]. First, it highlights the possibility that UBE2C-high CESC tumors represent a biologically distinct subtype characterized not only by aggressive cell cycle dysregulation but also by a remodeled immune landscape. Second, this linkage may partly explain the heterogeneous responses of cervical cancer patients to immunotherapeutic strategies, including immune checkpoint blockade [54].

From a therapeutic perspective, UBE2C has emerged as a potential target due to its tumor-restricted overexpression and essential role in mitotic progression [55,56]. Inhibition of UBE2C or disruption of the APC/C complex has been shown to suppress tumor growth and induce mitotic catastrophe in preclinical models [57]. Although such approaches primarily target tumor cell–intrinsic pathways, their impact on the tumor immune microenvironment (TIME) warrants further investigation. It is conceivable that attenuating tumor cell proliferation could indirectly modulate macrophage recruitment or functional polarization, thereby enhancing anti-tumor immune responses.

Nevertheless, several limitations of the current study should be acknowledged. Our findings are primarily based on transcriptomic and spatial association analyses, which reveal a robust correlation between UBE2C-high regions and macrophage infiltration (Fig. 2 and Fig. 3). However, these spatial coincidences do not yet establish a direct causal relationship. To provide initial mechanistic support for this link, our functional validation (Supplementary Fig. S1E) demonstrated that shRNA-mediated knockdown of UBE2C directly modulates the mRNA expression of key inflammatory mediators, including a prominent upregulation of IL10. This suggests that UBE2C levels can influence the secretable cytokine profile, which may in turn impact the surrounding immune landscape. Ultimately, while the present study utilizes high-resolution spatial transcriptomics to map the UBE2C-associated niche, the absence of *in vivo* animal models remains a limitation. Future investigations employing PDX or xenograft models, combined with complex co-culture systems, will be essential to definitively determine whether UBE2C-driven proliferative programs actively orchestrate macrophage behavior. Such studies are required to further validate the systemic antitumor efficacy and safety of Topotecan in targeting the UBE2C-associated immunosuppressive microenvironment in CESC.

## 5 Conclusion

**Figure 8:**
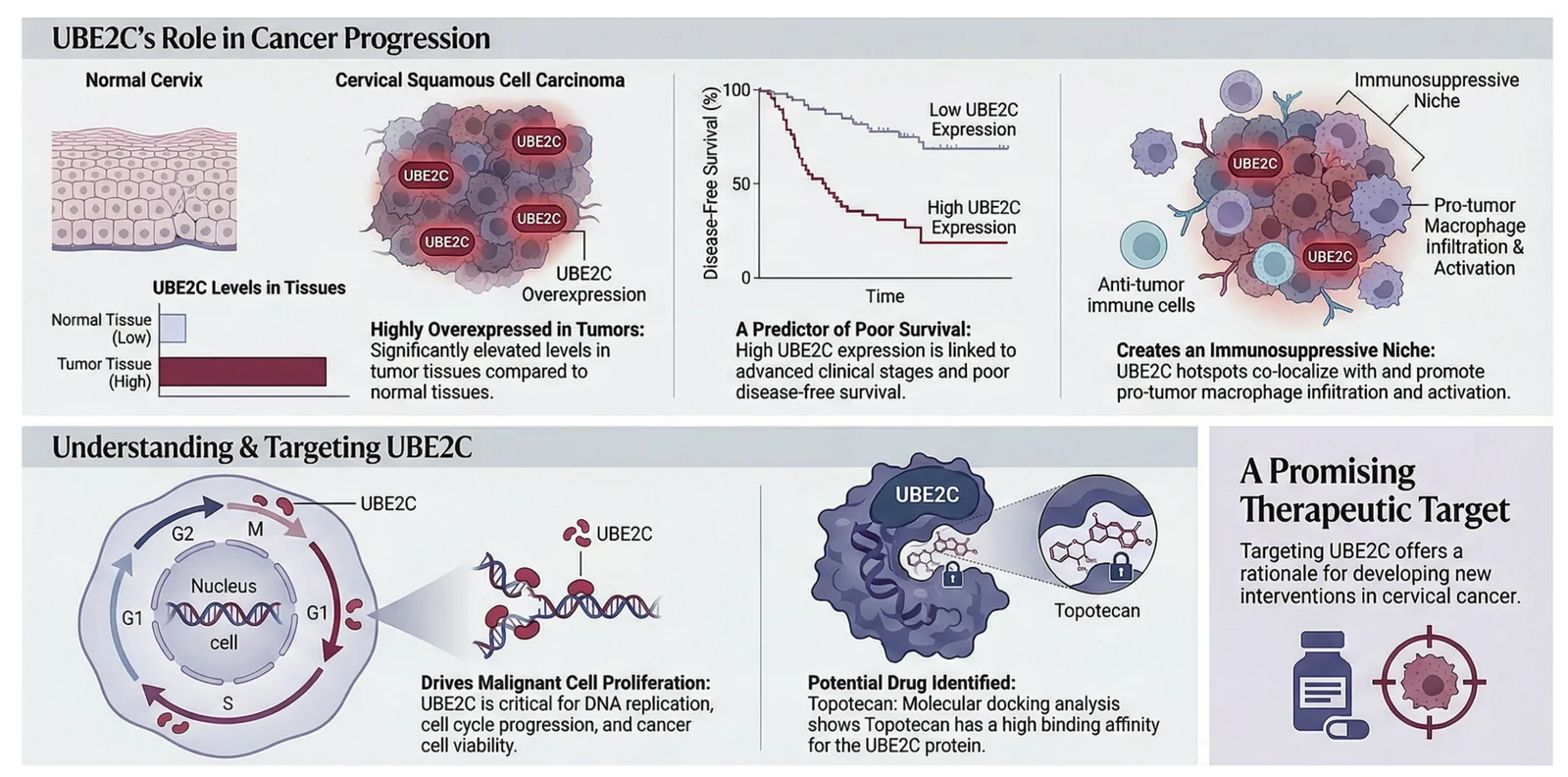
**Graphical summary of the clinical significance and oncogenic role of UBE2C in the cervical cancer immune microenvironment.** UBE2C overexpression correlates with advanced stages and poor prognosis, driving an immunosuppressive niche via macrophage infiltration. Essential for DNA replication and cell cycle regulation, UBE2C represents a critical therapeutic target. Topotecan is identified as a potential inhibitor with high binding affinity for UBE2C, providing a novel approach to remodel the tumor microenvironment (TME). Schematic components of this figure were created with BioRender.com.

In summary, our findings demonstrate that UBE2C is significantly overexpressed in CESC and positively correlates with an immune microenvironment characterized by M2/TAM-associated macrophage enrichment. While our results confirm UBE2C’s primary role in regulating DNA replication and cell cycle progression within tumor cells, the observed association with specific immune landscape features suggests its potential involvement in tumor progression and therapeutic response. These findings underscore the necessity of integrating tumor-intrinsic proliferative programs with the immune contexture in cervical cancer. Overall, this study identifies UBE2C as a promising candidate for further functional validation as a biomarker and therapeutic target (Fig. 8).

## Data Availability

Data openly available in a public repository.

## Supplementary Materials

The supplementary material is available online at https://www.techscience.com/doi/10.32604/or.2026.079551/s1.

## Abbreviations

**CESC**	Cervical squamous cell carcinoma and endocervical adenocarcinoma
**TAMs**	Tumor-associated macrophages
**TME**	Tumor microenvironment
**TIME**	Tumor immune microenvironment
**GEO**	Gene Expression Omnibus
**TCGA**	The Cancer Genome Atlas
**GTEx**	Genotype-Tissue Expression
**DEG**	Differentially expressed gene
**UMAP**	Uniform manifold approximation and projection
**GSEA**	Gene set enrichment analysis
**ssGSEA**	Single-sample gene set enrichment analysis
**FDR**	False discovery rate
**UBE2C**	ubiquitin conjugating enzyme E2C
