# Supplementary material for: Multi-Omics Identification of UBE2C as a Prognostic Biomarker and Therapeutic Target Linked to Topotecan Sensitivity in Cervical Cancer
Source: Oncol Res. 2026 Aug 13;34(9):18. doi: 10.32604/or.2026.079551 (PMC13494477; doi:10.32604/or.2026.079551)
Supplement: Supplementary file 1 [file OncolRes-34-79551-s001.zip › TSP_OR_79551-s001.docx]

**Supplementary Figure S1: UBE2C correlates with cytokine signaling and macrophage migration pathways in CESC.** (A), Correlation heatmap and (B), scatter plots illustrating the relationship between UBE2C expression and various cytokines, chemokines, and growth factors, highlighting a significant positive association with *VEGFB*. (C), Comparison of various Gene Ontology (GO) functional scores related to immune responses, inflammatory pathways, and cytokine production between UBE2C-high and UBE2C-low patient groups. (D), Box plots comparing GO function scores for macrophage migration and chemotaxis regulation between high and low UBE2C expression groups. Statistical significance was determined by appropriate correlation and comparative tests as indicated in the panels. (E), Quantitative PCR analysis showing the relative mRNA expression of various cytokines and chemokines, including *CCL5, CXCR4, IL6, IL8,* and *IL10*, following shRNA-mediated knockdown of *UBE2C*. * p<0.05.

**Supplementary Figure S2:** Transcriptomic and pharmacogenomic analysis of Topotecan response in UCEC. (A), Ridge plot illustrating significantly enriched biological pathways in UCEC cell lines following treatment with Topotecan, highlighting the suppression of cell cycle and mitotic processes. (B–E), GSEA plots demonstrating the negative enrichment of gene sets related to cell cycle mitosis (B), M phase (C), G2/M phases (D), and G2/M checkpoints (E), in Topotecan-treated UCEC cells. (F), Correlation analysis between UBE2C mRNA expression levels and Topotecan IC50 values across uterine cancer cell lines from the GDSC database. (G), Heatmap showing the expression patterns of various UBE2 family members in Topotecan-sensitive versus resistant UCEC cell lines. (H), Volcano plot and (I), heatmap illustrating the differentially expressed genes (DEGs) identified between Topotecan-sensitive and resistant groups. (J), Multiple gene correlation bubble plot representing the polygenic associations between UBE2C and core cell cycle/DNA replication regulators in Topotecan-treated UCEC cells. (K), Heatmap displays the expression profiles of key cell cycle markers, including UBE2C, CCNB1, and CDC20, across sensitive and resistant UCEC cell lines. Statistical significance for all comparisons and correlations was determined using appropriate Wilcoxon rank-sum or Spearman correlation tests.

**Supplementary Figure S3: Topotecan-induced RNA expression levels of UBE2C, CCNB1, CDC25C, and CDC20 in ovarian cancer cell lines.** (A-P), Representative box plots and heatmap analysis illustrating the changes in UBE2C expression in response to Topotecan treatment, demonstrating concentration- (A,B), and time-dependent (C,D), downregulation across various ovarian cancer cell lines. Box plots and heatmaps depicting the inhibitory effects of Topotecan on CCNB1 expression over different incubation periods (E, F), and drug concentrations (G, H). Box plots and heatmap analysis showing the concentration- (I, J), and time-dependent (K, L), suppression of CDC25C levels following Topotecan exposure. Box plots and heatmaps summarizing the concentration- (M, N), and time-dependent (O, P), decrease in CDC20 mRNA expression across the ovarian cancer cell line panel. Data are presented as log2 fold change compared to blank controls, with statistical significance and fold-change values indicated within the respective panels. The red box highlighted in the box plot indicates the inhibitory effects of topotecan in ovarian cancer cell lines (NCIADRRES, SKOV3, OVCAR8, OVCAR5, OVCAR4, NIHOVCAR3 and IGROV1).

**Supplementary Figure S4: Analysis of Topotecan-induced transcriptomic changes and gene correlations in urinary tract cancer.** (A), Cross-association scatter plot identifying significantly upregulated (red) and downregulated (blue) RNA transcripts in urinary tract cancer cell lines following Topotecan treatment. (B, C), Functional enrichment analysis performed via Metascape and DisGeNET databases, revealing key cellular pathways, such as DNA replication and RNA splicing (B), and disease associations linked to the observed RNA changes (C). (D), Box plots illustrating mRNA expression levels of ANAPC13, CCNL2, CDC25A, CDK11A, and DFFB in relation to Topotecan sensitivity (expressed as -log IC50). Statistical significance was determined by p-values indicated within each panel.

**Supplementary Figure S5: Impact of UBE2C and UBE2K genetic perturbations on gene expression and drug sensitivity in urinary tract cancer.** (A-D), Evaluation of the relationship between UBE2C knockdown efficacy (sgRNA efficacy) and the transcript levels of immune-related markers. (A), Correlation scatter plot showing a positive correlation with IL15RA expression in UBE2C knockdown efficacy in urinary tract cancer cell lines. (B), Correlation box plot showing a positive correlation with IL15RA expression. (C), Correlation scatter plot showing a significant negative correlation with TNFRSF1B levels in urinary tract cancer cell lines. (D), Correlation box plot showing a significant negative correlation with TNFRSF1B levels. (E), Cross-association correlation scatter plot correlating sgUBE2K efficacy with various drug responses, highlighting a prominent positive association with Topotecan sensitivity (red). (F), Correlation scatter plot confirming that higher sgUBE2K knockdown efficacy is significantly associated with increased sensitivity to Topotecan (expressed as -log IC50) in urinary tract cancer cell lines. (G), Correlation box plot confirming that higher sgUBE2K knockdown efficacy is significantly associated with increased sensitivity to Topotecan (expressed as -log IC50). Statistical associations were determined using Spearman correlation coefficients, fold-change values, and p-values as indicated in the respective panels.

**Supplementary Figure S6: Genomic perturbation and cell cycle signature analysis of Topotecan response in UCEC.** (A), Gene Set Enrichment Analysis (GSEA) plot evaluating gene sets associated with UBE2C upregulation ("UBE2C Up") in UCEC cell lines following Topotecan treatment. (B) GSEA plot evaluating gene sets associated with UBE2C downregulation ("UBE2C Down") in UCEC cell lines following Topotecan treatment. (C) Correlation scatter plot illustrating the relationship between transcriptomic signatures derived from UBE2C genetic perturbations (D21051_UBE2C_UP) and Topotecan IC50 values across UCEC cases in the GDSC database. (D), Correlation scatter plot illustrating the relationship between transcriptomic signatures derived from UBE2C genetic perturbations (D21051_UBE2C_Down) and Topotecan IC50 values across UCEC cases in the GDSC database. (E), Heatmap of ssGSEA scores depicting the enrichment of specific cell cycle phase signatures (G1, S, G2, G2/M) across Topotecan-sensitive and resistant uterine cancer cell lines. (F), Correlation analysis between the Whitefield cell cycle G2 signature and Topotecan IC50 in UCEC cell lines. Statistical significance was determined using the Wilcoxon rank-sum test or Spearman correlation, with p-values and R-values indicated within the respective panels.
